# NTCP modeling and dose–volume correlations for acute xerostomia and dry eye after whole brain radiation

**DOI:** 10.1186/s13014-021-01786-6

**Published:** 2021-03-21

**Authors:** Panayiotis Mavroidis, Kevin A. Pearlstein, Dominic H. Moon, Victoria Xu, Trevor J. Royce, Ashley A. Weiner, Colette J. Shen, Lawrence B. Marks, Bhishamjit S. Chera, Shiva K. Das, Kyle Wang

**Affiliations:** Department of Radiation Oncology, University of North Carolina, 101 Manning Dr., Chapel Hill, NC 27599-7512 USA

**Keywords:** Radiobiological parameters, Xerostomia, Dry eye, Whole brain radiation therapy, NTCP, LKB, Relative seriality

## Abstract

**Background:**

Whole brain radiation (WBRT) may lead to acute xerostomia and dry eye from incidental parotid and lacrimal exposure, respectively. We performed a prospective observational study to assess the incidence/severity of this toxicity. We herein perform a secondary analysis relating parotid and lacrimal dosimetric parameters to normal tissue complication probability (NTCP) rates and associated models.

**Methods:**

Patients received WBRT to 25–40 Gy in 10–20 fractions using 3D-conformal radiation therapy without prospective delineation of the parotids or lacrimals. Patients completed questionnaires at baseline and 1 month post-WBRT. Xerostomia was assessed using the University of Michigan xerostomia score (scored 0–100, toxicity defined as ≥ 20 pt increase) and xerostomia bother score (scored from 0 to 3, toxicity defined as ≥ 2 pt increase). Dry eye was assessed using the Subjective Evaluation of Symptom of Dryness (SESoD, scored from 0 to 4, toxicity defined as ≥ 2 pt increase). The clinical data were fitted by the Lyman–Kutcher–Burman (LKB) and Relative Seriality (RS) NTCP models.

**Results:**

Of 55 evaluable patients, 19 (35%) had ≥ 20 point increase in xerostomia score, 11 (20%) had ≥ 2 point increase in xerostomia bother score, and 13 (24%) had ≥ 2 point increase in SESoD score. For xerostomia, parotid V_10Gy_–V_20Gy_ correlated best with toxicity, with AUC 0.68 for xerostomia score and 0.69–0.71 for bother score. The values for the D_50_, m and n parameters of the LKB model were 22.3 Gy, 0.84 and 1.0 for xerostomia score and 28.4 Gy, 0.55 and 1.0 for bother score, respectively. The corresponding values for the D_50_, γ and s parameters of the RS model were 23.5 Gy, 0.28 and 0.0001 for xerostomia score and 32.0 Gy, 0.45 and 0.0001 for bother score, respectively. For dry eye, lacrimal V_10Gy_–V_15Gy_ were found to correlate best with toxicity, with AUC values from 0.67 to 0.68. The parameter values of the LKB model were 53.5 Gy, 0.74 and 1.0, whereas of the RS model were 54.0 Gy, 0.37 and 0.0001, respectively.

**Conclusions:**

Xerostomia was most associated with parotid V_10Gy_–V_20Gy_, and dry eye with lacrimal V_10Gy_–V_15Gy_. NTCP models were successfully created for both toxicities and may help clinicians refine dosimetric goals and assess levels of risk in patients receiving palliative WBRT.

## Background

Whole brain radiation (WBRT) is a common treatment for patients with brain metastases [1–6]. Many patients who receive WBRT have a poor prognosis, and it is important to minimize both acute and late toxicities. Commonly-acknowledged consequences of WBRT include neurocognitive effects, fatigue, and hair loss [7–9].

More-recently, in a prospective study, we reported that patients receiving standard WBRT (without prospective delineation of the parotid or lacrimal glands) resulted in clinically-significant acute xerostomia and dry eye in roughly 35% and 25% of cases, respectively with toxicity rates associated with glandular doses [1, 10]. In those reports, a summary of the reported toxicities was provided together with a statistical analysis of their correlation against given dose volume metrics. However, as there are no prior studies reporting NTCP model parameter values for these acute toxicities, we performed additional dosimetric analyses including normal tissue complication probability (NTCP) modelling. Different groups in research and clinical domain are more familiar with and prone to use a given NTCP model. For this reason, in this study, parameter values were derived for two popular NTCP models in order to facilitate such groups incorporate NTCP metrics for the examined toxicities in their analyses. Also, dose thresholds were identified in this study, which seeks to generate risk assessment tools to aid in clinical decision making.

## Methods

### Patient selection, treatment and OAR delineation

Patients were treated with WBRT on a prospective, IRB-approved study (ClinicalTrials.gov #NCT02682199), with details of study procedures previously reported [1, 10]. In brief, patients were eligible if they were planned to receive WBRT for any indication using 3D planning to a total dose of 25–40 Gy in 10–20 fractions, at 2–3 Gy per fraction. Patients provided written consent and were enrolled at one academic center and two affiliated community hospitals.

All patients were treated in the supine position using customized head-cast immobilization. Patients were treated using 3D planning without prospective delineation of the parotid glands or lacrimal glands, though some providers delineated the globe and/or lens for avoidance. Using digitally-reconstructed radiographs, WBRT fields were designed based on bony anatomy and covered the entire skull and extended to the inferior border of the C1 or C2 vertebrae [1].

The bilateral parotid and lacrimal glands were retrospectively delineated without knowledge of clinical outcomes. Parotids were delineated using planning CT alone, whereas MRI fusion was used for lacrimal gland delineation. The dose volume histograms (DVH) were calculated for the bilateral parotid and lacrimal glands. DVH-based metrics were correlated with patient reported outcomes and definitions of toxicity as described below.

### Definition of toxicity for xerostomia and dry eye

Patients completed xerostomia and dry eye questionnaires at baseline pre-WBRT, at the conclusion of WBRT, and at 1, 3, and 6 months post-WBRT. All toxicity analyses refer to outcomes at 1 month (1M) post-WBRT, which was the prospectively-specified primary time point. To allow NTCP modelling, clinically significant toxicity was defined as a binary variable using threshold worsening of patient-reported symptom scores, as described below.

For xerostomia, patients completed the validated University of Michigan Xerostomia Questionnaire (xerostomia score), calculated using eight questions each scored from 0 to 10 and linearly converted to a 100-point scale, with higher scores representing worse symptoms [11–13]. Patients also completed a 2-question xerostomia bother score that assessed the degree to which xerostomia bothered patients while eating and while not eating [11]. Each bother question was answered on a 4-point Likert scale (0: Bothered not at all, 1: Bothered a little bit, 2: Bothered quite a bit, and 3: Bothered very much) adapted from the EORTC QLQ-H&N35 head and neck cancer-specific QOL questionnaire [11, 14]. The higher score of the two bother questions was considered the overall bother score at that time point. Two definitions of clinically significant toxicity were used for the xerostomia analysis: (1) ≥ 20 point increase in xerostomia score and (2) ≥ 2 point increase in xerostomia bother score [11]. Regarding those two scores, as it has been reported in the literature and has been confirmed by our data, lower levels of increase from baseline were characterized by large variability. However, for the levels that we used in this study the responses were more stable over time constituting a clear worsening of the symptoms from baseline. The results and analysis of xerostomia using bother score are presented in the “Appendix”.

For dry eye, patients completed the Subjective Evaluation of Symptoms of Dryness (SESoD) [1, 15–17]. The SESoD is a single question assessing the presence and significance of dry eye and is scored on a 5-point Likert scale (0: None, 1: Minimal, 2: Mild, 3: Moderate, 4: Severe).

Clinically significant toxicity was defined as a ≥ 2 point increase in SESoD score [1].

### Radiobiological models

Physical dose distributions were converted to equivalent doses of 2 Gy per fraction (EDQ_2Gy_) using an α/β value of 3 Gy [18–20]. For each organ, the corresponding generalized equivalent uniform doses were calculated (gEUD_2Gy_) [21, 22]. The clinical data relating to the observed rate of NTCP was used to calculate the model parameters for both the Lyman–Kutcher–Burman (LKB) model [23, 24] and the relative seriality (RS) [25]. The basic parameters of each model are: *D*_50_ (or TD_50_), which is the dose for a complication rate of 50%, the slope (gradient) of the dose response curve (*m* for LKB and γ for RS), and the parameter that accounts for the volume dependence of the organ (*n* for LKB and *s* for RS). From the formulation of the RS model, the biologically effective uniform dose ($\overline{\overline{D}}$) is derived. $\overline{\overline{D}}$ is the dose that causes exactly the same normal tissue complication probability as the real dose distribution [26]. The mathematical formulations of NTCP models and biological doses are provided in the “Appendix”. In the figures and tables presenting results of the NTCP models, equivalent to 2 Gy per fraction doses are used otherwise the physical doses are shown.

### Statistical methods

For the two NTCP models, the parameter values and their 95% confidence intervals were determined using the maximum likelihood method [18, 27, 28]. The fitting calculations were performed through the use of a minimization package (MINOS) [29]. The confidence intervals of the model parameters were determined using the profile likelihood method. The ability of the NTCP models to distinguish patients with and without the examined symptoms was evaluated using the area under the curve (AUC) measure, which is used as a summary of the ROC curve [18, 30]. The goodness-of-fit of the different NTCP models was assessed through the Hosmer–Lemeshow test [31]. Additionally, the Odds Ratio (OR) method was applied to identify NTCP thresholds beyond which the risk of toxicity increases significantly [18, 32]. Those thresholds were identified in three steps. First, we identified the thresholds for which the OR values are larger than 1 and sorted them by OR value (largest to lowest); second, we identified the thresholds for which the low limit of the 95% confidence interval (95% CI) is larger than one; and third, we identified the threshold with the smallest 95% CI.

## Results

100 patients were enrolled and treated between 2015 and 2018, of whom 55 and 54 were eligible for the analyses of 1-month xerostomia and dry eye, respectively (45 patients were prospectively excluded from analysis due to lack of baseline score or baseline SESoD score ≥ 3, or did not complete WBRT, or did not complete any follow-up questionnaires at 1 month post-RT). Patient characteristics are shown in Table 1.

**Table 1:** Patient characteristics (n = 55) [1]

Patient characteristics		
Age, median (range), y	61 (23–82)	
Primary diagnosis		
Breast cancer	9	16%
Lung cancer	37	67%
Melanoma	4	7%
Other	5	9%
ECOG PS		
0	17	31%
1	27	49%
2	9	16%
3	2	4%
On baseline steroids	31	56%
On baseline narcotics	24	44%
On baseline anticholinergics	24	44%
Post-RT chemo before 1 month	29	53%
Fractionation scheme		
2.5 Gy × 10 = 25 Gy	3	6%
3 Gy × 10 = 30 Gy	32	58%
2 Gy × 15 = 30 Gy	1	2%
2.5 Gy × 14 = 35 Gy	17	31%
2 Gy × 20 = 40 Gy	2	4%

*ECOG PS* Eastern Cooperative Oncology Group Performance Status, *RT* radiation, *Gy* gray

Most patients received 30 Gy in 10 fractions (58%) or 35 Gy in 14 fractions (31%). For the xerostomia analysis, clinically significant toxicity was observed in 19 patients (35%) who had a ≥ 20 point increase in xerostomia score. For the dry eye analysis, clinically significant toxicity was observed in 13 patients (24%) who had a ≥ 2 point increase in SESoD score.

**Fig. 1:**
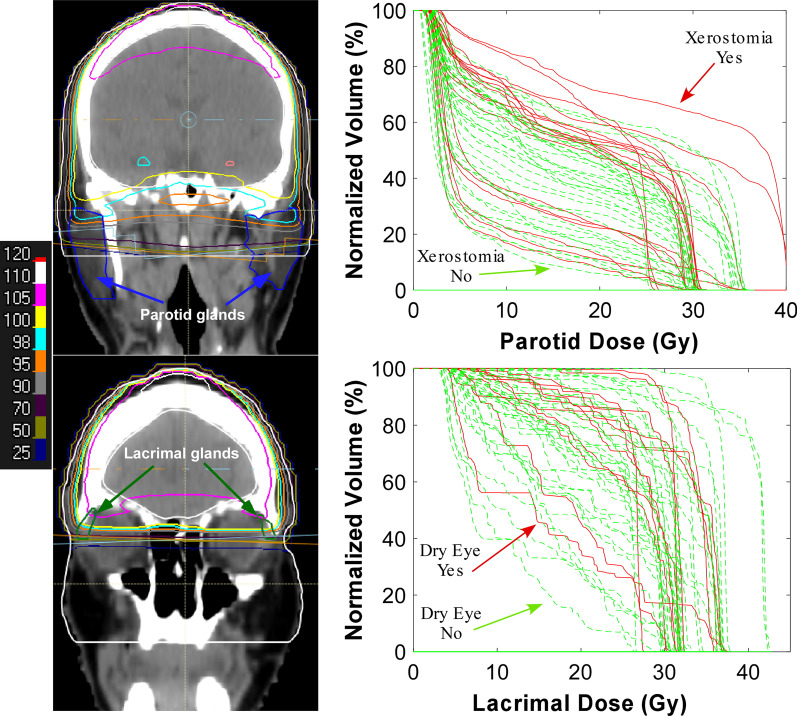
Upper*:* the spatial dose distribution of a representative patient and DVHs of the combined parotid glands for patients with (red solid lines) versus without (green dotted lines) clinically significant xerostomia defined using xerostomia score (≥ 20 point worsening in xerostomia score). Lower*:* the spatial dose distribution of a representative patient and DVHs of the combined lacrimal glands for patients with versus without clinically significant dry eye (≥ 2 point worsening in SESoD score). The isodose lines represent percentages of the prescription dose (30 Gy)

**Fig. 2:**
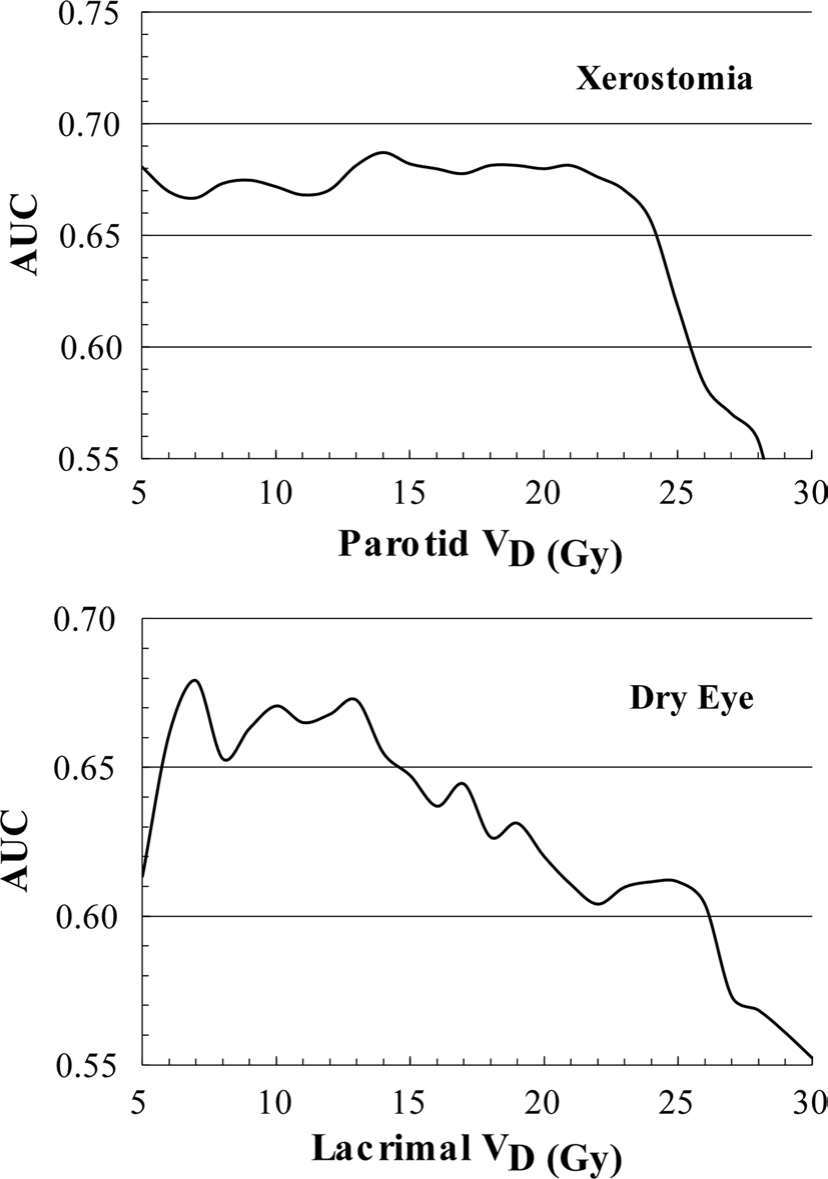
Upper*:* AUC curves of the combined parotid glands for clinically significant xerostomia. Lower*:* AUC curves of the combined lacrimal glands for clinically significant dry eye. The x-axis refers to the dose (D) of the dose volume metric (V_D_) and has units of Gy

Figure 1 shows a coronal view of the spatial dose distribution for a representative patient in the plans of the parotid and lacrimal glands, respectively. It also illustrates the bilateral parotid and lacrimal dose volume histograms (DVHs) for patients with and without toxicity. Table 2 presents average OAR mean and volumetric dose in patients with and without toxicity. Figure 2 shows the areas under the ROC curves (AUC) for different parotid and lacrimal dose volume metrics for their corresponding toxicity endpoints.

**Table 2:** Average dosimetric parameters for patients with and without toxicity

Endpoint	Xerostomia	
	Toxicity	No toxicity
Number of patients	19	36
Parotid mean ± std. dev. (Gy)	16.2 ± 6.2	13.8 ± 4.7
Parotid V_20_ ± std. dev. (%)	43.0 ± 19.5	34.6 ± 15.7

Endpoint	Dry eye	
	Toxicity	No toxicity
Number of patients	13	41
Lacrimal mean ± std. dev. (Gy)	26.6 ± 4.9	25.0 ± 6.7
Lacrimal V_15_ ± std. dev. (%)	86.3 ± 15.7	78.3 ± 19.7

*std. dev.* standard deviation

For xerostomia, the AUC values for D_mean_ and V_20Gy_ of the parotid glands were 0.64 and 0.68, respectively. Patients with parotid V_20Gy_ ≥ 49% had 8.6 (95% CI: 2.4–30.8) times higher risk for clinically significant toxicity as defined using xerostomia score (this cutoff was determined using the Odds Ratio method).

For dry eye, the AUC value for D_mean_ to the lacrimal glands was 0.60, whereas the dose–volume indices in the range V_6Gy_–V_15Gy_ were found to correlate best with toxicity with AUC values ranging between 0.65 and 0.68. Patients with lacrimal V_15Gy_ ≥ 80% had a 5.8 (95% CI 1.1–29.4) times higher risk for clinically significant toxicity (this cutoff was determined using the Odds Ratio method).

**Fig. 3:**
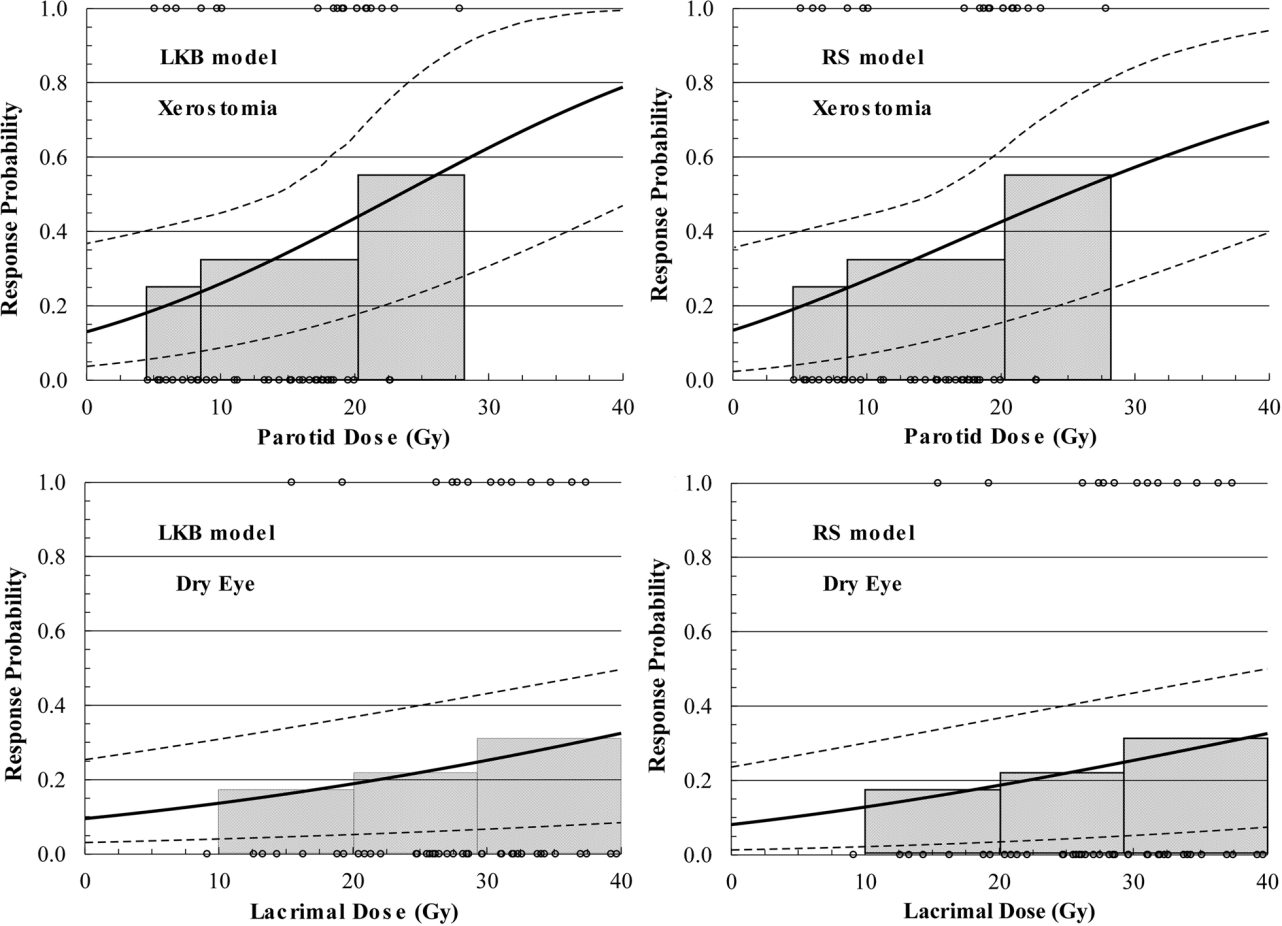
Upper*:* dose response curves for the parotid glands and clinically significant xerostomia. Lower*:* dose response curves for the lacrimal glands and clinically significant dry eye. The dashed lines correspond to the 95% confidence intervals of the dose response curves. The individual binary response data are shown as open circles. The doses on the x-axis correspond to gEUD and $\overline{\overline{D}}$ (for the LKB and RS models, respectively). The shaded histograms represent the response rates as a function of mean dose to the parotid or lacrimal glands, respectively

Results for NTCP modelling parameters using LKB and RS methods are shown in Table 3, for xerostomia and dry eye, respectively. Figure 3 shows the parotid and lacrimal dose response curves generated using these models, for a range of gEUD and $\overline{\overline{D}}$ doses (for the LKB and RS models, respectively). Overall results for the NTCP modelling, AUC analysis, and assessment of statistical significance are summarized in Table 4 for xerostomia and dry eye, respectively. The goodness of fit of the NTCP models was evaluated by the Hosmer–Lemeshow test, which showed that the *p* values of the LKB and RS models were 0.35 for xerostomia and 0.52 for dry eye, respectively. Both values are larger than 0.05, so the null hypothesis that the observed and expected response rates are the same across all doses cannot be rejected.

**Table 3:** Summary of the parameter values and 95% confidence intervals for the LKB and RS models of the parotid glands for the endpoint of xerostomia and lacrimal glands for dry eye

Parameters	LKB model		
	*D*_50_ (Gy)	*m*	*n*
Xerostomia	23.4 (16.4–41.8)	0.89 (0.56–2.96)	1.0 (0.6–1.0)
Dry eye	61.2 (40.6–153.0)	0.77 (0.54–1.51)	1.0 (0.1–1.0)

Parameters	Relative seriality model		
	*D*_50_ (Gy)	γ	*s*
Xerostomia	24.9 (16.5–48.2)	0.26 (0.00–0.49)	10^–4^ (10^–5^ to 7 × 10^–4^)
Dry eye	63.9 (39.9–142.9)	0.34 (0.14–0.54)	10^–4^ (10^–5^ to 7 × 10^–4^)

**Table 4:** Summary of the results from the fit of the two normal tissue complication probability models for xerostomia and dry eye. AUC = area under the curve. Response rate is the average of the response rates predicted by the models. OR = Odds ratio. Threshold doses refer to gEUD and $\overline{\overline{D}}$ (for the LKB and RS models, respectively). The *p* value was calculated using the two sample t-test for the patient subgroups above and below the dose threshold (the null hypothesis is that the true mean difference is zero)

Parameters	Response rate (%)	AUC	Odds ratio		*p* value
			Threshold (Gy)	OR (95% CI)	
*LKB model*					
Xerostomia	34.7	0.67	20	5.6 (1.5–20.6)	0.03
Dry eye	24.1	0.57	27	2.6 (0.6–10.9)	0.09
*Relative seriality model*					
Xerostomia	34.6	0.67	20	5.8 (1.5–23.2)	0.03
Dry eye	24.1	0.56	27	2.6 (0.6–10.9)	0.09

## Discussion

In this analysis, we expanded on our recent reports [1, 10] by creating NTCP models and refining the dosimetric relationships linking parotid dose to xerostomia and lacrimal dose to dry eye in patients receiving WBRT. The dose–volume metrics V_13Gy_–V_23Gy_ and V_6Gy_–V_15Gy_ of the parotid and lacrimal glands were best correlated with the endpoints of xerostomia and dry eye, respectively (see Fig. 2). The AUC values of those dose metrics are close to the AUC values of D_mean_ for both organs (parotid and lacrimal glands). This is because they demonstrate a parallel-like volume effect against the endpoints of xerostomia and dry eye, respectively. In both cases, the respective V_20_ and V_15_ metrics showed higher AUC values but not at a statistically significant level.

The OR values that are reported here are the highest values, which are also statistically significant (lower limit of the 95% CI should be larger than one) and have the have the smallest confidence interval. However, it has to be stated that the accompanied thresholds depend on the cohort characteristics (e.g. distribution of dose values among patients). Those results have an immediate clinical applicability since they can be used as dose constraints in treatment plan optimization and evaluation.

In WBRT, the vast majority of patients are treated with parallel-opposed lateral fields. IMRT/VMAT are not well-accepted approaches since the cost and planning/QA time for IMRT/VMAT are far higher than for opposed laterals, and given the clinical setting, might not be justified. In order to establish a closer dose–response relation for dry eye, the response of the individual eyes could be recorded. However, in most cases the status of the patient is not good. Getting good QOL data is challenging and asking questions about each eye separately would add mental burden on the patients.

For xerostomia, our findings are consistent with recent reports from radiotherapy of head and neck tumors finding that parotid V_20_ correlates best with PRO-CTCAE scores [33]. For example, the difference in parotid mean dose between patients with vs. without xerostomia was only 2.4 Gy, whereas the difference in parotid V_20Gy_ was 8.4% (Table 2). A statistically significant threshold of V_20Gy_ ≤ 47% corresponded to an odds ratio of 8.6 (95% CI 2.4–30.8).

For dry eye, there are no good prior studies for comparison. More specifically, although there are a couple of studies performing NTCP modeling for lacrimal glands, neither of those use the LKB and RS models or have the same or a similar clinical endpoint [e.g. Bhandare et al. use a different model (Logit) and a different toxicity endpoint (ophthalmologic diagnosis of severe DES)] [34]. In our analysis, the same pattern was found as with xerostomia in that volumetric dose metrics were also better correlated with toxicity than mean doses. For example, in our group, the mean lacrimal dose difference between patients with vs. without dry eye was only 1.6 Gy, whereas the difference in lacrimal V_15Gy_ was 8%. A statistically significant threshold of V_15Gy_ ≤ 80% corresponded to an odds ratio of 4.4 (95% CI 1.3–15.4).

There are many studies reporting NTCP model parameters for xerostomia after head and neck radiotherapy. The values of the NTCP model parameters we computed for the same endpoint but after WBRT are not very different than those reported in the literature. When the later ones were applied to the present dataset many of them were found compatible producing similar AUC and OR values with the fitted model parameters. The reason is that the previously published model parameter values fall within the confidence intervals of the derived values (see Tables 3, 5). For the parameter sets that were not found compatible, there are several potential reasons to explain this difference. First, most prior reports address patients with head and neck cancer, and the character of the 3D dose distribution is very different in the setting of WBRT. More specifically, dose fall off inside the volume of parotids is more pronounced in the case of head and neck radiotherapy.

**Table 5:** Summary of the parameter values that have been reported by different groups for the LKB model, regarding the endpoint of xerostomia. The difference between the *physician-scored* (CTCAE) *patient-reported* (PRO-CTCAE) scoring systems is also indicated. Those model parameter sets were applied on the current dataset and the corresponding AUC, OR (with 95% confidence interval and dose threshold) values were calculated

Parameters	*D*_50_ (Gy)	*m*	*n*	AUC	OR (95% CI)|thres
*CTCAE*					
Burman et al. [36]	46.0	0.18	0.7	0.53	0.0 (–)|–
Roesink et al. [37]	39.0	0.45	1.0	0.66	5.1 (1.1–23.4)|21
Braam et al. [38]	42.0	0.37	1.0	0.66	8.0 (1.8–35.6)|20
*PRO-CTCAE*					
Mavroidis et al. [35]	21.9	0.78	1.0	0.67	5.8 (1.5–23.2)|20

Second, most prior studies have used *physician-scored* toxicity (CTCAE), while the current study uses PRO [35–38]. It is generally understood that providers might *underestimate* the rate/severity of toxicities. In this light, the differences between our results and the prior studies (see Table 5) are logical as lower values for *TD*_50_, and higher values for *m*, and higher values for *n* would be associated with predicting a *higher* rate of toxicity (as our use of PROs would be expected to yield higher risk rates). Interestingly, our computed parameters are close to parameters derived from our recent analysis of *patient-reported* symptoms (PRO-CTCAE) in patients with head and neck cancer as presented in Table 5 [33]. This agreement suggests that the models might be reasonably applicable across a broad range of 3D-dose/volume characters.

Third, most prior studies have considered the dose/volume metrics for the *contralateral* parotid alone (since there is usually/often no attempt made to spare the ipsilateral parotid gland), where we considered *both* parotids as a pooled single structure in the setting of WBRT.

We found that both the LKB and RS NTCP models were able to be fitted to the clinical xerostomia data (Table 4), with similar goodness-of-fit. Indeed, the threshold doses for xerostomia in both NTCP models were identical (18 Gy). This is similar to what has been found in other studies where both models provide a reasonable fit to the data [23, 35].

The results of xerostomia based on bother score have very similar pattern with those based on Michigan Questionnaire. More specifically, both scoring systems have almost the same V_20Gy_ and Odds ratio statistics. However, the NTCP model values show some deviation (*TD*_50_/*D*_50_ values are higher and *m*/γ values are lower for bother score).

For dry eye, there is minimal published dose/volume data/modelling results to which we can compare our findings. As with the parotids, the threshold doses for dry eye were similar for the two models (28 Gy for the gEUD in the LKB and 25 Gy for the $\overline{\overline{D}}$ in the RS). Although the data show that there an increased risk for dry eye beyond those threshold doses, the OR results were not statistically significant. This means that a more conservative approach should be followed regarding the use of those threshold doses in the clinic.

In this study, serial observations were collected (from 1 to 6 months) aiming at analyzing the dependence of velocity and extent of symptom resolution on dose. Unfortunately, the follow-up data at 3 and 6 months are sparse to allow a valid statistical analysis. More specifically, the available follow-up data were 55, 33 and 28 at 1, 3 and 6 months, respectively.

Our study has several limitations. First, we did not consider provider-defined toxicity scoring. Nevertheless, we think our approach is reasonable as there is increasing recognition of the importance of patient-reported outcomes [35]. Second, the definition of a significant toxicity was somewhat subjective (e.g., a threshold symptom score increase from baseline). However, this a common approach that has been used by other groups using patient-reported outcomes, and it is reasonable since it inherently acknowledges the importance of considering baseline status [35]. This is particularly important in our setting since many patients with brain metastases requiring WBRT would have received prior systemic therapies that might impact these symptoms. Third, the total number of patients and events was modest. A rule of thumb in modelling is to have 10 events per parameter in the dataset. When this rule breaks, the results show large confidence intervals. However, in our case this is reasonable as the study was prospective, and to our knowledge this is one of the largest studies of its kind to address this issue in the setting of WBRT.

Finally, it is known that all the existing NTCP models have inherent limitations. These models do not account for biological mechanisms (cell redistribution, re-oxygenation, etc.) that may have an impact on treatment outcome. Further, such models do not consider the spatial distribution of dose, and hence ignore the possibility that there may be particularly-important regions of such “parallel organs” in determining clinical response [39]. Despite these uncertainties, the correlations of the model-based NTCP values with clinical outcome data were fairly good.

## Conclusions

In conclusion, we created NTCP models that reasonably-well fit acute parotid and lacrimal gland toxicity after WBRT. These models and their findings may help establish levels of risk for these acute toxicities and help clinicians determine reasonable dosimetric goals to maintain quality of life in patients receiving palliative WBRT. Further investigations are needed to refine/confirm these model-based estimates.

## Figures and Tables

**Fig. 4:**
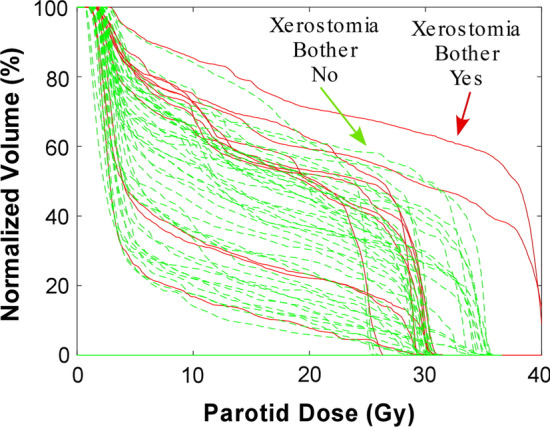
The DVHs of the combined parotid glands for patients with (red solid lines) versus without (green dotted lines) clinically significant xerostomia defined using the bother score (right, ≥ 2 point worsening in bother score)

**Fig. 5:**
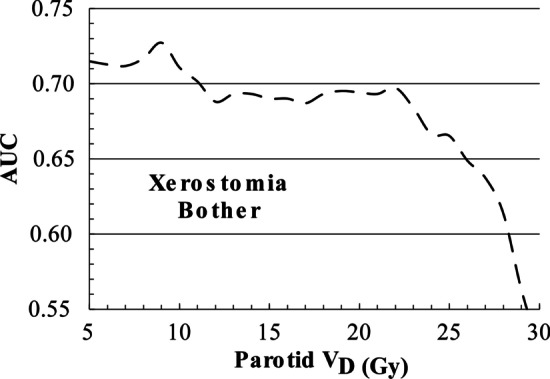
Upper: AUC curves of the combined parotid glands for clinically significant xerostomia as defined using the bother score

**Fig. 6:**
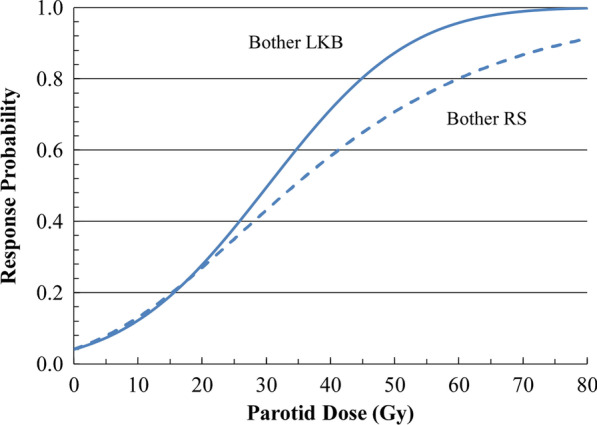
Upper*:* Dose response curves for the parotid glands and clinically significant xerostomia using the bother score. The solid line correspond to the LKB model and dashed line correspond to the RS model. The doses on the x-axis correspond to gEUD and $\overline{\overline{D}}$ (for the LKB and RS models, respectively)

## Data Availability

The datasets used and/or analyzed during the current study are available from the corresponding author on reasonable request.

## Appendix

### Analysis using bother score for xerostomia

Figure 4 shows bilateral parotid dose volume histograms (DVHs) for patients with and without toxicity based on bother score. Table 6 presents average parotid mean and volumetric dose in patients with and without toxicity. Figure 5 shows the areas under the ROC curves (AUC) for different parotid dose volume metrics for xerostomia using bother score. Table 7 presents a summary of the parameter values and 95% confidence intervals for the LKB and RS models of the parotid glands for the endpoint of xerostomia using the bother score and Fig. 6 schematically illustrates the corresponding dose response curves.

**Table 6:** Average dosimetric parameters for patients with and without toxicity

Endpoint	Xerostomia bother score	
	Toxicity	No toxicity
Number of patients	11	44
Parotid mean ± std. dev. (Gy)	17.2 ± 6.7	14.0 ± 4.8
Parotid V_20_ ± std. dev. (%)	45.5 ± 19.4	35.5 ± 16.5

*std. dev.* standard deviation

**Table 7:** Summary of the parameter values and 95% confidence intervals for the LKB and RS models of the parotid glands for the endpoint of xerostomia using the bother score

Parameters	LKB model		
	*D*_50_ (Gy)	*m*	*n*
Xerostomia bother score	30.2 (22.3–50.6)	0.58 (0.40–0.96)	1.0 (0.6–7.0)

Parameters	Relative seriality model		
	*D*_50_ (Gy)	γ	*s*
Xerostomia bother score	34.3 (24.0–61.4)	0.42 (0.23–0.61)	10^–4^ (10^–5^ to 7 × 10^–4^)

Table 8 presents a summary of the results from the fit of the two normal tissue complication probability models for xerostomia using the bother score. Clinically significant toxicity was observed in 11 patients (20%) who had a ≥ 2 point increase in xerostomia bother score. The AUC values for D_mean_ and V_20Gy_ of the parotid glands were 0.65 and 0.69, respectively. Patients with parotid V_20Gy_ ≥ 49% had 9.1 (95% CI 2.0–40.7) times higher odds of clinically significant toxicity as defined using bother score. The goodness-of-fit of the NTCP models was evaluated by the Hosmer–Lemeshow test, which showed that the *p* values of the LKB and RS models were 0.31 and 0.30, respectively for the xerostomia bother score. This value is larger than 0.05, so the null hypothesis that the observed and expected response rates are the same across all doses cannot be rejected.

**Table 8:** Summary of the results from the fit of the two normal tissue complication probability models for xerostomia using the bother score. AUC = area under the curve. Response rate is the average of the response rates predicted by the models. OR = Odds ratio. The *p* value was calculated using the two sample t-test for the patient subgroups above and below the dose threshold (the null hypothesis is that the true mean difference is zero)

Parameters	Response rate (%)	AUC	Odds ratio		*p* value
			Threshold (Gy)	OR (95% CI)	
*LKB model*					
Xerostomia bother score	20.1	0.69	20	5.3 (1.2–22.9)	0.009
*Relative seriality model*					
Xerostomia bother score	20.0	0.68	19	6.8 (1.6–28.4)	0.001

### NTCP model fitting validation using bootstrapping

In order to further validate the NTCP fitting results, an internal sensitivity analysis was performed using bootstrapping. More specifically, we created 1000 bootstrap (with replacement) samples from the original dataset and applied the fitted NTCP model parameters. Then, we calculated the AUC and OR values for each sample. Finally, we calculated the average values and 95% confidence intervals. Also, using those 1000 bootstrap samples we performed a similar analysis for the V_20_ and V_15_ dose volume metrics for xerostomia and dry eye, respectively. The results of the analysis are shown in Tables

**Table 9:** Bootstrap results for the fitted dose volume metrics for xerostomia and dry eye. AUC = area under the curve. Response rate is the average of the response rates predicted by the models. OR = Odds ratio. The unit of threshold is percentage

Parameters	AUC (95% CI)	OR (95% CI)	Threshold (95% CI)
*V*_*20*_			
Xerostomia-Michigan	0.68 (0.50–0.85)	7.5 (3.3–17.8)	52.3 (47.0–59.0)
Xerostomia-Bother	0.70 (0.48–0.90)	7.8 (2.6–15.3)	52.8 (48.0–61.0)
*V*_*15*_			
Dry eye	0.64 (0.46–0.82)	5.3 (1.7–11.0)	89.0 (82.0–96.0)

9 and

**Table 10:** Summary of the results from the fit of the two normal NTCP models for xerostomia and dry eye. AUC = area under the curve. Response rate is the average of the response rates predicted by the models. OR = Odds ratio. Threshold represent values of gEUD for the LKB model and $\overline{\overline{D}}$ for the RS model

Parameters	AUC (95% CI)	OR (95% CI)	Threshold (95% CI)
*LKB model*			
Xerostomia-Michigan	0.67 (0.49–0.83)	7.5 (2.9–16.6)	20 (18–22)
Xerostomia-Bother	0.69 (0.47–0.89)	7.7 (2.1–15.4)	20 (17–23)
Dry eye	0.56 (0.39–0.74)	3.9 (1.0–10.0)	29 (25–35)
*RS model*			
Xerostomia-Michigan	0.67 (0.49–0.84)	7.1 (3.0–16.0)	20 (18–22)
Xerostomia-Bother	0.69 (0.46–0.89)	7.4 (1.9–15.4)	20 (17–22)
Dry eye	0.56 (0.39–0.73)	3.8 (1.0–10.0)	28 (25–35)

10.

## Abbreviations

WBRT: Whole brain radiation
NTCP: Normal tissue complication probability
LKB: Lyman–Kutcher–Burman
RS: Relative seriality
SESoD: Subjective evaluation of symptom of dryness
DVH: Dose volume histogram
3D: Three dimensional
CT: Computed tomography
MRI: Magnetic resonance imaging
RT: Radiation therapy
QOL: Quality of life
EDQ_2Gy_: Equivalent doses of 2 Gy per fraction
gEUD: Generalized equivalent uniform dose
$\overline{\overline{D}}$: Biologically effective uniform dose
*D*_50_: Dose for a complication rate of 50% (for the RS model)
TD_50_: Dose for a complication rate of 50% (for the LKB model)
γ: Slope (gradient) of the dose response curve (for the RS model)
m: Slope (gradient) of the dose response curve (for the LKB model)
s: Parameter that accounts for the volume dependence of the organ (for the RS model)
m: Parameter that accounts for the volume dependence of the organ (for the LKB model)
IMRT: Intensity modulated radiotherapy
VMAT: Volumetric modulated arc therapy
AUC: Area under the curve
ROC: Receiver operating characteristic
OR: Odds ratio
PRO: Patient-reported outcomes
CTCAE: Common Terminology Criteria for Adverse Events
